# Enhancing Amplification in Compliant Mechanisms: Optimization of Plastic Types and Injection Conditions

**DOI:** 10.3390/polym16030394

**Published:** 2024-01-31

**Authors:** Pham Son Minh, Van-Thuc Nguyen, Tran Minh The Uyen, Vu Quang Huy, Hai Nguyen Le Dang, Van Thanh Tien Nguyen

**Affiliations:** 1 Faculty of Mechanical Engineering, Ho Chi Minh City University of Technology and Education, Ho Chi Minh City 71307, Vietnam; minhps@hcmute.edu.vn (P.S.M.); nvthuc@hcmute.edu.vn (V.-T.N.);; 2 Faculty of Mechanical Engineering, Industrial University of Ho Chi Minh City, Nguyen Van Bao Street, Ward 4, Go Vap District, Ho Chi Minh City 70000, Vietnam

**Keywords:** ABS, HDPE, PP, amplification ratio, filling time, packing time

## Abstract

This study surveys the impacts of injection parameters on the deformation rate of the injected flexure hinge made from ABS, PP, and HDPE. The flexure hinges are generated with different filling time, filling pressure, filling speed, packing time, packing pressure, cooling time, and melt temperature. The amplification ratio of the samples between different injection parameters and different plastic types is measured and compared to figure out the optimal one with a high amplification ratio. The results show that the relationship between the input and output data of the ABS, PP, and HDPE flexure hinges at different injection molding parameters is a linear relation. Changing the material or many injection molding parameters of the hinge could lead to a great impact on the hinge’s performance. However, changing each parameter does not lead to a sudden change in the input and output values. Each plastic material has different optimal injection parameters and displacement behaviors. With the ABS flexure hinge, the filling pressure case has the greatest amplification ratio of 8.81, while the filling speed case has the lowest value of 4.81. With the optimal injection parameter and the input value of 105 µm, the ABS flexure hinge could create a maximum average output value of 736.6 µm. With the PP flexure hinge, the melt temperature case achieves the greatest amplification ratio of 6.73, while the filling speed case has the lowest value of 4.1. With the optimal injection parameter and the input value of 128 µm, the PP flexure hinge could create a maximum average output value of 964.8 µm. The average amplification ratio values of all injection molding parameters are 6.85, 5.41, and 4.01, corresponding to ABS, PP, and HDPE flexure hinges. Generally, the ABS flexure hinge has the highest amplification ratios, followed by the PP flexure hinge. The HDPE flexure hinge has the lowest amplification ratios among these plastic types. With the optimal injection parameter and the input value of 218 µm, the HDPE flexure hinge could create a maximum average output value of 699.8 µm. The results provide more insight into plastic flexure hinges and broaden their applications by finding the optimal injection parameters and plastic types.

## 1. Introduction

Recently, flexure hinges have been intensively investigated for precision actuation due to their complexity and low cost [1,2,3,4]. Flexure hinges work as a displacement amplification mechanism with no friction force and backlash; therefore, they can replace the conventional system such as motors in super precision machines in lithography, space telescopes, microelectromechanical systems, and optical devices [5,6,7,8]. Some metals and alloys are usually stainless steel, titanium-based, copper-based, and aluminum alloys to create a flexure hinge [9,10,11,12]. The metal-based alloy flexure hinges provide good elastic properties and high corrosion resistance. For example, Wei et al. [13] created a SUS 316L stainless steel hinge from 3D printing. The report showed that the porous layer thickness of the hinge is like the average diameter of the powder particle. Coemert et al. [14] generated Ti-6Al-4V flexure hinges via laser cutting. The results indicated that the payload value is proportional to the hinge width but inversely proportional to the length. Interestingly, Roopa et al. [15] studied the impacts of geometry and material on the displacement of stainless steel, beryllium copper, and brass flexure hinges. They pointed out that the deflection values of the flexure hinge are 6.75 µm, 10.89 µm, and 12.10 µm, corresponding to stainless steel, beryllium copper, and brass materials. Schlick et al. [16] reported an aluminum gripper based on a flexure hinge mechanism. The gripper was applied as a micro-assembly station, and the momentum can be limited by controlling the oscillation via the actuator, lowering the gripping jaw speed, and moving coil actuators.

Flexure hinges with compliant mechanisms could have many designs, depending on the purposes, such as displacement amplifiers, smart structures, robotics, and quasi-static mechanisms. Typically, compliant flexure hinges for displacement amplifier applications have many types such as bridge type, Rhombus type, differential amplifier, lever mechanism, five bar structure, and tensural types. Compared to other designs, a bridge-type flexure hinge has the advantages of a simple and symmetrical shape, high load capacity, and high amplification ratio. Generally, flexure hinges are usually created using metals. However, fabricating a flexure hinge from these material types often requires a lot of time and cost because of the complicated structure and the high precision of the hinge. Using alternative materials with better productivity in fabricating flexure hinges is a rigorous demand. For example, Mutlu et al. [17] used TPE to produce a 3D printing flexure hinge and pinpointed that the elliptic and non-symmetric shapes have the best quality. Rosa et al. [18] created a flexure-based nanopositioner using cyclic olefin copolymer (COC) via the mesoscale injection molding process. The travel range of the COC nanopositioner is 15 µm with the highest standard deviation being 52.3 nm. Notably, Sahota et al. [19] simulated a fiber Bragg grating pressure sensor by comparing ABS polymer and stainless steel flexural hinges. The simulation results showed that the sensitivity of the ABS flexural hinge is 16 times higher than that of the stainless steel one. Shen et al. [20] combined bridge type and lever type mechanisms to generate a hybrid one, achieving a high amplification ratio and low overstress. Abedi et al. [21] designed a bridge-type compliant mechanism with an S-shape, achieving an amplification ratio of 4.5–5.5. Shi et al. [22] created a microgripper with two bridge-type parallelogram amplification mechanisms, obtaining a high amplification ratio of 30.3. Chen et al. [23] used a hybrid-compliant mechanism with a bridge-type and a lever-type design to harvest the vibration energy. Wan et al. [24] optimized the design process of the flexure-based bridge-type amplification using a reliability-based design optimization method. This method provided good accuracy compared to the other traditional methods. Overall, plastic flexural hinge investigations are not popular and need more insight.

Injection molding is a popular technique that could produce plastic parts at a mass production level due to its advantages of industrial availability, high accuracy, short time, and affordable cost [25,26,27,28]. Injection molding, therefore, is widely applied in generating household products and industrial parts. Moreover, advanced injection molding techniques such as microinjection molding, gas-assisted molding systems, conformal cooling channels, metal injection molding, foam injection molding, and water-assisted injection molding have received much interest from authors [29,30,31]. Injection molding can be applied with many thermoplastic polymers such as HDPE, LDPE, PP, ABS, and PA [32,33,34,35]. However, the reports about plastic flexure hinges generated using the injection molding process are rarely discussed despite the advantages of this fabrication technique.

This investigation focuses on creating plastic bridge-type mechanism flexure hinges with a leaf hinge from different plastic types including ABS, PP, and HDPE. The injection parameters including filling time, filling pressure, filling speed, packing time, packing pressure, cooling time, and melt temperature are also surveyed to optimize the hinge performance. The amplification ratio of the samples is evaluated and compared across several injection parameters and plastic types to determine the optimal one with a high amplification ratio. The results provide more insight into plastic flexure hinges and broaden their applications by finding the optimal injection parameters and plastic types.

## 2. Experimental Methods

**Figure 1:**
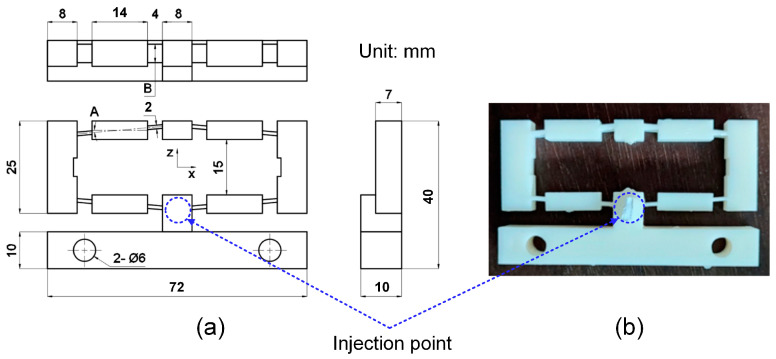
Plastic bridge-type flexure hinge: (**a**) design and (**b**) injected hinge [36].

**Figure 2:**
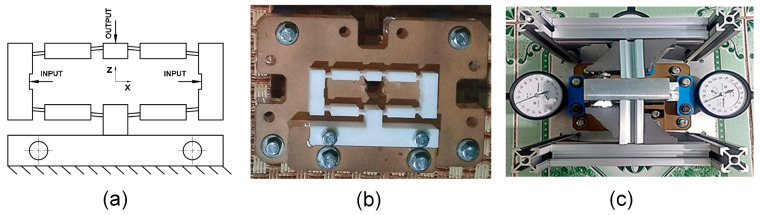
Compliant mechanism of the flexure hinge, fixture, and measurement device: (**a**) compliant mechanism, (**b**) fixture, and (**c**) measurement device setup [36].

**Figure 3:**
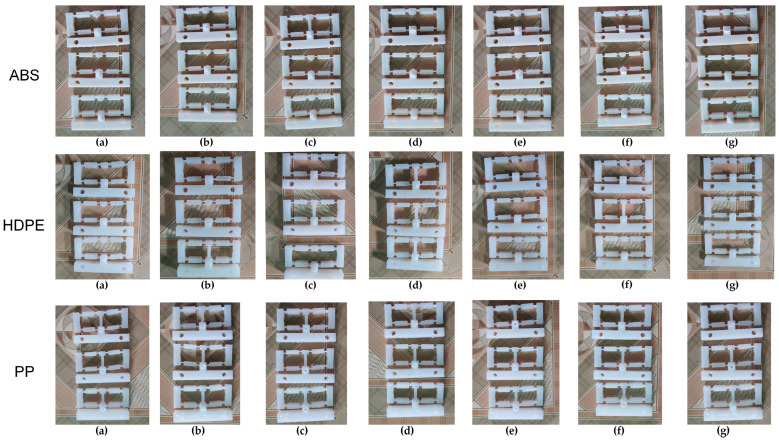
The flexure hinges at different plastics and injection conditions: (**a**) cooling time, (**b**) filling pressure, (**c**) filling speed, (**d**) filling time, (**e**) melt temperature, (**f**) packing pressure, and (**g**) packing time.

Figure 1 shows the flexure hinge design and the injected hinge. The injection gate dimension is 5 mm in diameter. The compliant mechanism of the flexure hinge with input and output position, the fixture of the hinge, and the measurement device with chronographs for measuring the displacement of the hinge are presented in Figure 2. The measurement procedure consists of three steps. Step 1: Attach the sample to the bracket in the proper position and then attach the chronographs. Step 2: Align the pusher with the measuring model and adjust it. All chronographs had to be reset to zero. Step 3: Turn the pusher handle, then watch the chronographs and record the results. For each sample number, the study injected three plastic hinges for an individual measurement. The average number of three samples is calculated and presented in this report. The standard deviation varies by around 1–7%. Figure 3 presents the flexure hinge at different plastics and injection conditions. Besides holding time and holding pressure, the study also applied packing time and packing pressure. They are the extra steps of the injection molding cycle in which pressure is applied to the polymer melt to compress the polymer and drive additional material into the mold. This compensates for shrinkage as the polymer cools from the melt temperature to the room temperature. The gate is closed during the packing operation to prevent material from entering the mold. Moreover, the molding part weight is about 12–13.7 g, which is suitable for the holding and cooling time.

After the setup of the experiment machines, the plastics are dried at 85 °C for 12 h to remove the humidity. Then, they are injected into the mold using an injection molding. Finally, the injection flexure hinge is tested using a fixture and measurement device. This machine has a screw diameter of 36 mm, an injection capacity of 157 g, a screw speed of 122 mm/s, a 3 mm nozzle with a temperature limit switch control, and a clamping force of 1200 kN. Injection molding parameters depend on the geometric features of the moldings (especially thickness), the flow path of the polymer melt, the number of injection points, the type of polymeric material, the injection machine, etc. During the initial step, which is the experimental setup, this study tried many injection parameters for different plastic types to ensure that the samples were successfully injected. Firstly, this study attempted with relatively low parameters, in which the samples were partially injected. An attempt to inject the molded part was made by gradually increasing the values of the parameters. After that, the injection parameters are gradually adjusted to improve the injection quality. Finally, after having enough information, the injection parameters are set up as shown in Table 1 and Table 2. The injection machine in this report is MA 1200III (Haitian, China). The weights of the moldings are 12 g, 12.7 g, and 13.7 g, corresponding to the PP, HDPE, and ABS hinges.

The injection rate of the screw is 154 cm^3^/s. The filling speed is presented based on the number “154 cm^3^/s”. The percentage presents the ratio between the selected filling speed and the number “154 cm^3^/s”. Regarding the mold temperature, this study does not apply a mold temperature assistance system. Therefore, the mold temperature could be 30 °C. These hinges are injected with different injection molding parameters and different plastics. Table 1 shows the injection mold parameters of the ABS hinge, while Table 2 illustrates the injection molding parameters of the PP and HDPE hinges. The filling time is set at 1.5–2.8 s because the study did not apply the maximum filling pressure and filling speed of the injection molding machine MA 1200III. Therefore, it requires more time to fill the mold cavity than the maximum condition. The injection pressure is quite low; however, it is enough to fill the mold cavity with a suitable filling time (1.5–2.8 s). Because the study did not use the injection molding machine MA 1200III’s maximum filling pressure and filling speed, the filling speed is set from 50% to 70%. As a result, a higher filling speed is required to fill the mold cavity than under the maximum condition. The study applied only 75–83% of this number, and according to the moderate pressure and volume of the runner system, the filling time must be greater than 2.0 s to ensure a good filling stage. Moreover, the subsequent packing stages (or holding stages) will ensure the product is filled with melt during the cooling phase, as shown in Table 1.

ABS 750 SW polymer is supplied by Kumho Petrochemical, Seoul, Republic of Korea. PP polymer named Advanced-PP 1100 N is manufactured by Advanced Petrochemical Company, Al Jubail, Saudi Arabia. HDPE polymer HTA 108 is manufactured by Exxon Mobile Petroleum and Chemical Company, Riyadh, Saudi Arabia. The Young’s modulus of ABS, PP, and HDPE are 3110 MPa, 1550 MPa, and 1300 MPa, respectively.

## 3. Results and Discussion

### 3.1. ABS Flexure Hinge

In this section, the effects of injection parameters on the displacement of the ABS flexure hinge are examined. Firstly, the impacts of the filling stage, including filling time, filling pressure, and filling speed, are surveyed.

**Figure 4:**
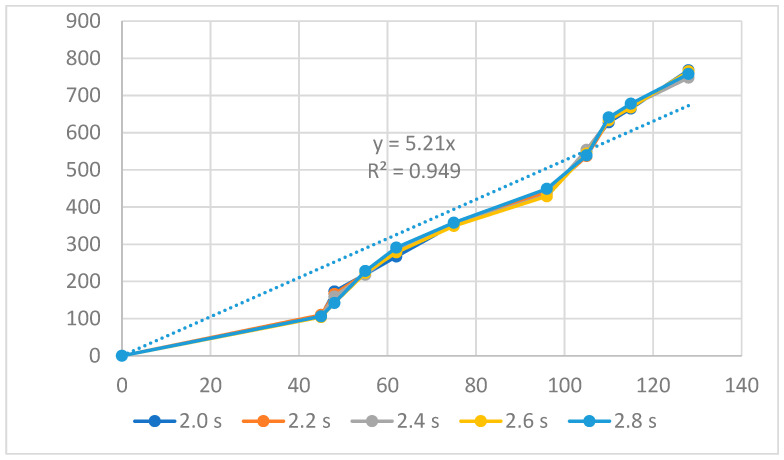
Displacement of the ABS flexure hinge at different filling times.

**Figure 5:**
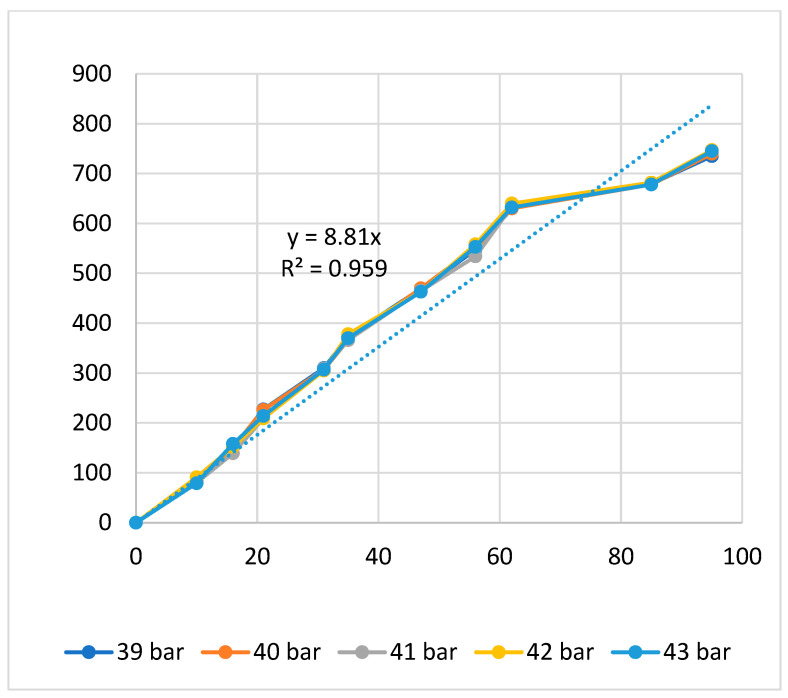
Displacement of the ABS flexure hinges at different filling pressures.

**Figure 6:**
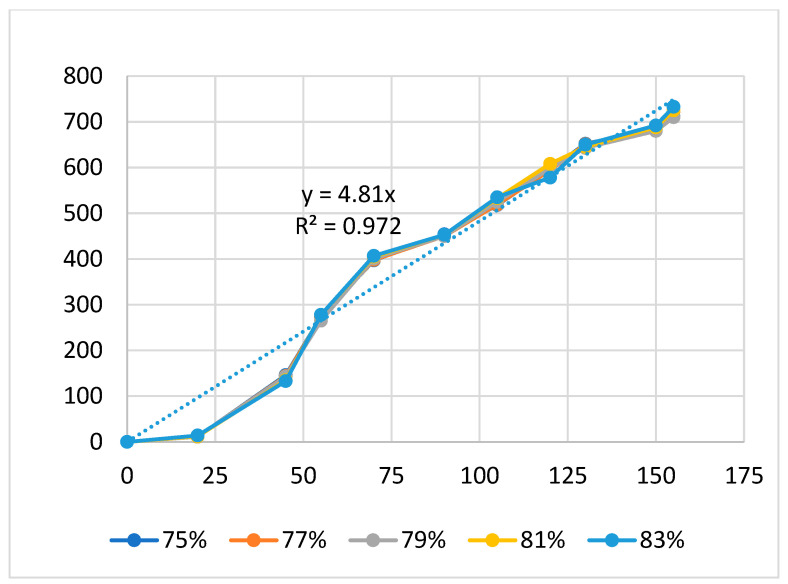
Displacement of the ABS flexure hinges at different filling speeds.

Figure 4, Figure 5 and Figure 6 present the displacement diagrams of the ABS flexure hinge at different filling times, filling pressures, and filling speeds. In the filling time case, with the maximum input value of 128 µm, the average output value is 760.4 µm, indicating the magnification effect of the hinge, as shown in Figure 4. Generally, changing the filling time, filling pressure, and filling speed does not strongly affect the displacement rate of the ABS flexure hinge due to the similar values of the curves at different filling times and filling pressures. In addition, improving the input value mostly leads to a linear increase in the output value. The regression equations between the average values of the input and the output displacements are

*y*_1_ = 5.21*x*_1_, (1)

*y*_2_ = 8.81*x*_2_, (2)

*y*_3_ = 4.81*x*_3_, (3)

where *y*_1_, *y*_2_, and *y*_3_ are the output data and *x*_1_, *x*_2_, and *x*_3_ are the input data of the ABS flexure hinge at different filling times, filling pressures, and filling speeds.

The trendline diagrams are set as a linear relationship y = a.*x* + b, where “b” is zero because the intercept of the trendline is set at zero. Therefore, there is only the “a” regression coefficient in the equation y = a.*x*. Moreover, the R-squared value of these trendlines is very high, which is higher than 0.9, indicating that the equation has a good prediction value.

This equation indicates a linear relationship between the input and the output data, as mentioned above. The amplification ratio of the filling time case is 5.21, the filling pressure case is 8.81, and the filling speed is 4.81. These values mean that the filling pressure cases have the highest amplification ratio, indicating the strong effect of the filling pressure on the amplification ratio. On the contrary, the filling speed has the lowest amplification ratio, meaning a weaker effect rate than the filling pressure and the filling time.

**Figure 7:**
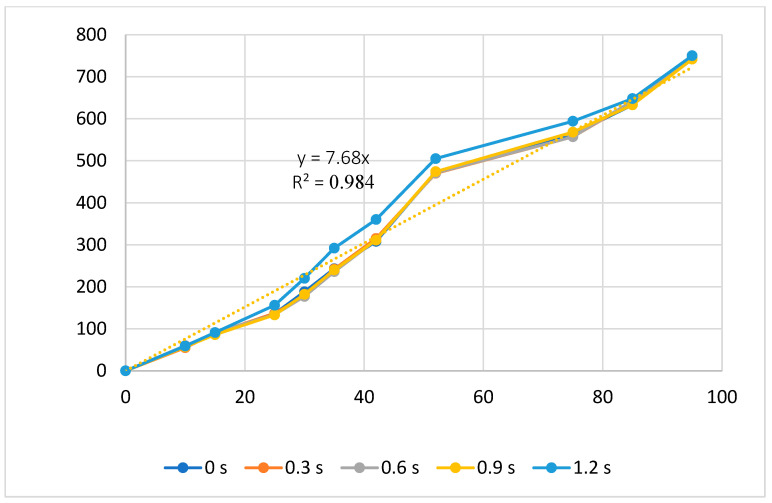
Displacement of the ABS flexure hinges at different packing times.

**Figure 8:**
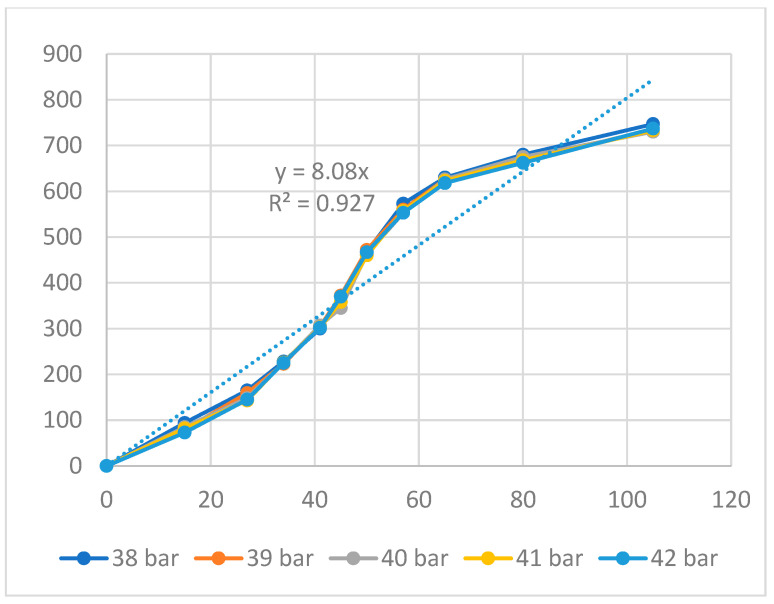
Displacement of the ABS flexure hinges at different packing pressures.

Figure 7 and Figure 8 present the displacement of the ABS flexure hinge at different packing times and packing pressures. Consistent with the filling stage results, the similar values at different packing times and packing pressures reveal that altering these values does not cause much change in the displacement rate. Moreover, the regression equations between the average values of the input and the output displacements are

*y*_4_ = 7.68*x*_4_, (4)

*y*_5_ = 8.08*x*_5_, (5)

where *y*_4_ and *y*_5_ are the output data and *x*_4_ and *x*_5_ are the input data of the ABS flexure hinge at different packing times and packing pressures.

These data have a linear relationship, which is similar to the filling stage. The amplification ratio values for the packing time and packing pressure cases are 7.68 and 8.08, respectively. These ratios are comparable, unlike the filling stage, where the filling pressure has a larger amplification ratio than the filling time.

**Figure 9:**
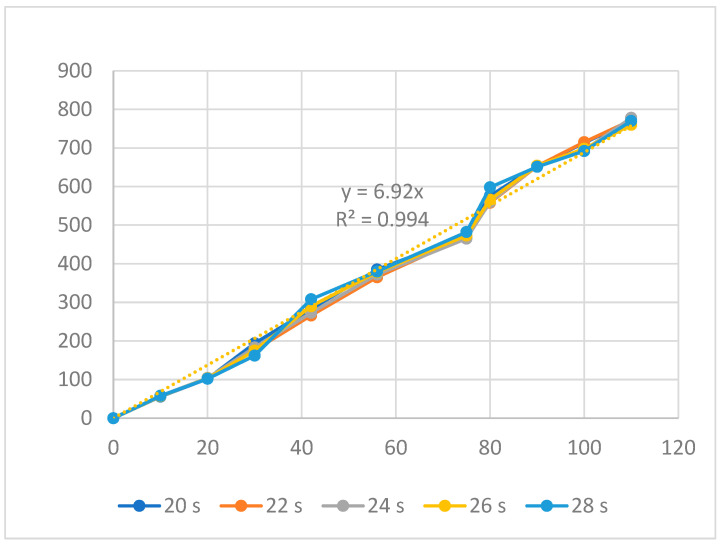
Displacement of the ABS flexure hinges at different cooling times.

**Figure 10:**
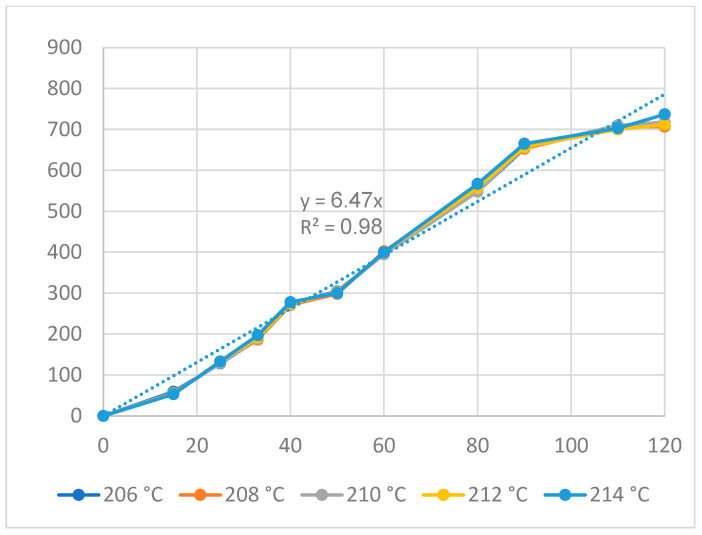
Displacement of the ABS flexure hinges at different melt temperatures.

Figure 9 and Figure 10 display the displacement of the ABS flexure hinge at different cooling times and melt temperatures. The curve diagrams for different parameters in every figure are almost equivalent, demonstrating that changing the cooling time and melt temperature has little effect on the displacement rate. Furthermore, the regression equations between the average values of the input and output displacements are as follows:

*y*_6_ = 6.92*x*_6_, (6)

*y*_7_ = 6.47*x*_7_, (7)

where *y*_6_ and *y*_7_ are the output data and *x*_6_ and *x*_7_ are the input data of the ABS flexure hinge at different cooling times and melt temperatures.

The linear relationship between the input and output data is revealed by Equations (6) and (7), which are similar to the filling and packing stages. The amplification ratio values for the cooling time and melt temperature cases are 6.92 and 6.47, respectively. The results reveal that the cooling time case has a higher amplification ratio, resulting in a higher effect rate.

Overall, the relationship between the input and output data of the ABS flexure hinges at different injection molding parameters is linear. The amplification ratio values for filling time, filling pressure, filling speed, packing time, packing pressure, cooling time, and melt temperature are 5.21, 8.81, 4.81, 7.68, 8.08, 6.92, and 6.47. The amplification ratio of filling pressure is the highest, while that of filling speed is the lowest. The range of the filling pressure examined is 39–43 bar. In this range, the amplification ratio is highest due to the good quality of the sample following the parameters of filling time 2.4 s, filling pressure 39–43 bar, packing time 0.6 s, packing pressure 40 bar, cooling time 24 s, melt temperature 210 °C, and filling speed 79%, while the filling speed range is 75–83%. In this range, the amplification ratio is lowest due to the lower quality of the sample following the parameters of filling time 2.4 s, filling pressure 41 bar, packing time 0.6 s, packing pressure 40 bar, cooling time 24 s, melt temperature 210 °C, and filling speed 75–83%.

Interestingly, the packing stage also shows high amplification ratio values of 7.68 and 8.08, pinpointing the important role of this stage in optimizing the ABS flexure hinge. Most importantly, the results reveal that the curve diagrams of each figure are mostly similar because changing each parameter does not lead to a sudden change in the input and output value. In general, the injection parameters strongly impact the amplification ratio of the ABS hinge. In other words, changing a set of injection parameters could lead to a greater impact on the hinge’s performance. The amplification ratio could range from 4.81 to 8.81, which is a relatively large deviation. The reason for this phenomenon is the sensitivity of the ABS flexure hinge performance when changing the injection parameters. The plastic injection hinge design with thin hinge corners especially requires suitable injection parameters to successfully fill the mold cavity because the plastic melt flow is hindered in these thin areas. The presented injection parameters are ideal for forming the ABS flexure hinge.

Overall, the amplification ratio of the ABS flexure hinge varies in the range of 4.81–8.81. In Kim et al.’s report [37], a similar aluminum alloy flexure hinge has an amplification of 5–25. The amplification ratio of the ABS hinge is lower than that of the aluminum alloy one. The reason is the higher strength and stiffness of the aluminum alloys compared to the ABS plastic.

### 3.2. PP Flexure Hinge

This section examines the influences of injection parameters on the displacement of the PP flexure hinge. In the previous section, the results reveal that the curve diagrams of each figure are mostly similar because changing each parameter does not lead to a sudden change in the input and output value. Furthermore, linear relationships are popular in all cases of the ABS flexure hinge. As a result, this section concentrates on the average value of each parameter. First, the impact of the filling stage is addressed, which includes filling time, filling pressure, and filling speed.

**Figure 11:**
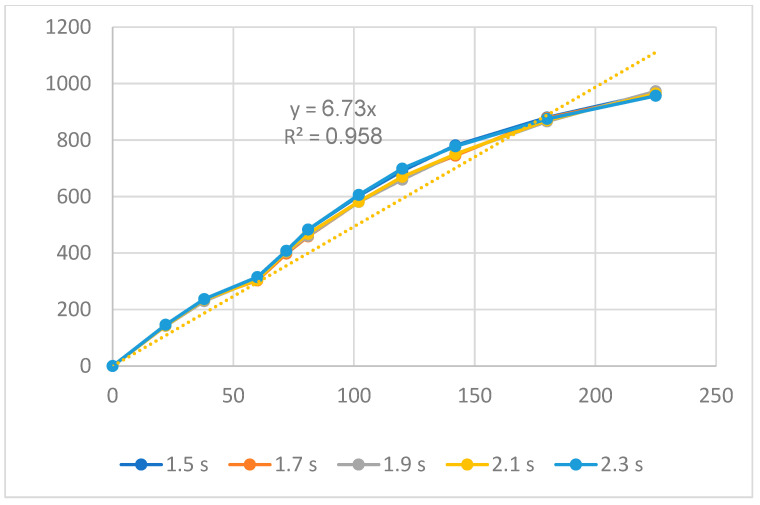
Displacement of PP flexure hinges at different filling times.

**Figure 12:**
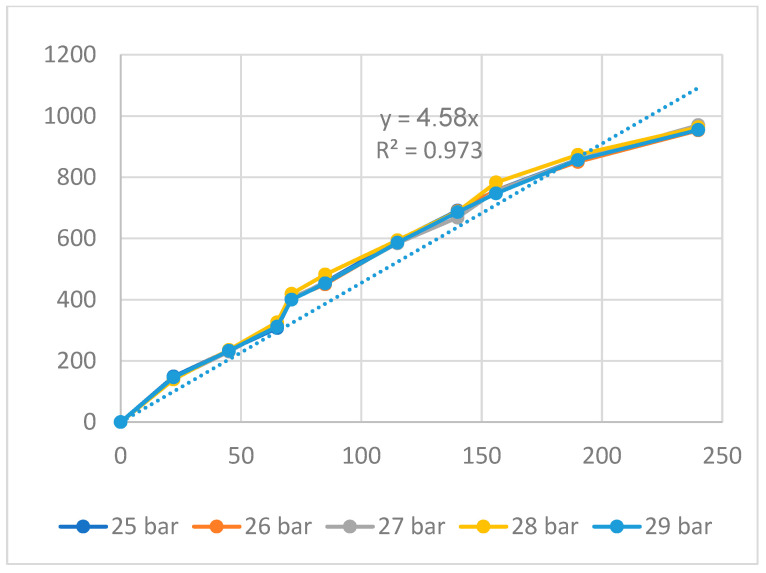
Displacement of PP flexure hinges at different filling pressures.

**Figure 13:**
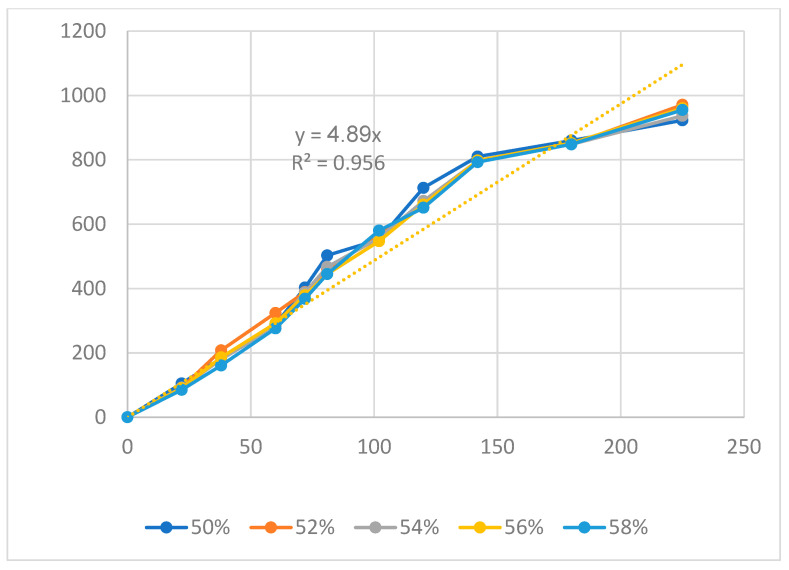
Displacement of PP flexure hinges at different filling speeds.

Figure 11, Figure 12 and Figure 13 display the displacement diagrams of the PP flexure hinge at different filling times, filling pressures, and filling speeds. Following calculations, the regression equations demonstrating the linear relationship between the average values of the input and output displacements are

*y*_8_ = 6.73*x*_8_, (8)

*y*_9_ = 4.58*x*_9_, (9)

*y*_10_ = 4.89*x*_10_, (10)

where *y*_8_, *y*_9_, and *y*_10_ are the output data and *x*_8_, *x*_9_, and *x*_10_ are the input data of the PP flexure hinge at different filling times, filling pressures, and filling speeds.

The amplification ratio of the filling time case is 6.73, the filling pressure case is 4.58, and the filling speed case is 4.89. Different from the ABS flexure hinge, in which the filling pressure has the highest amplification ratio, the filling time case achieves the highest amplification ratio of 6.83. On the other hand, the filling pressure case has the lowest amplification ratio of 4.58. Compared to the steel alloy flexure hinge with an amplification ratio of 16.2 in Na et al.’s report [38], the PP flexure hinge has a lower value. The reason is that steel alloy has a much higher elastic modulus and strength than PP plastic.

**Figure 14:**
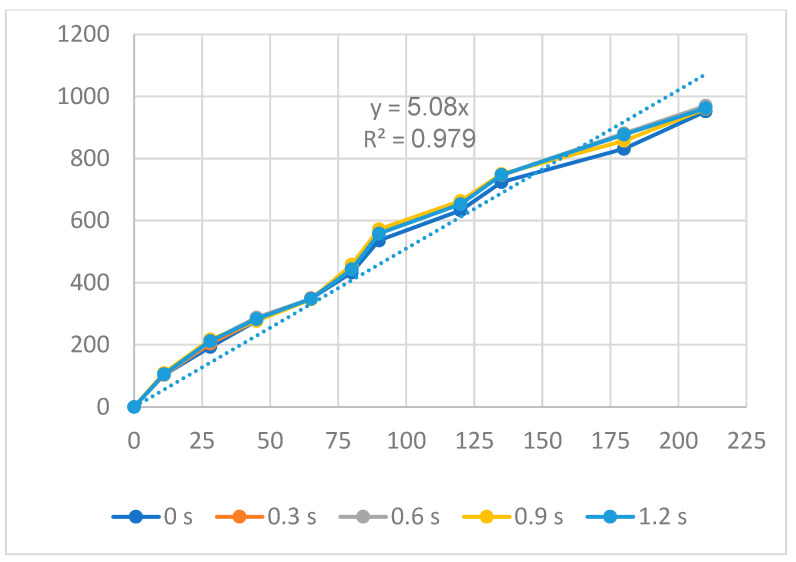
Displacement of PP flexure hinges at different packing times.

**Figure 15:**
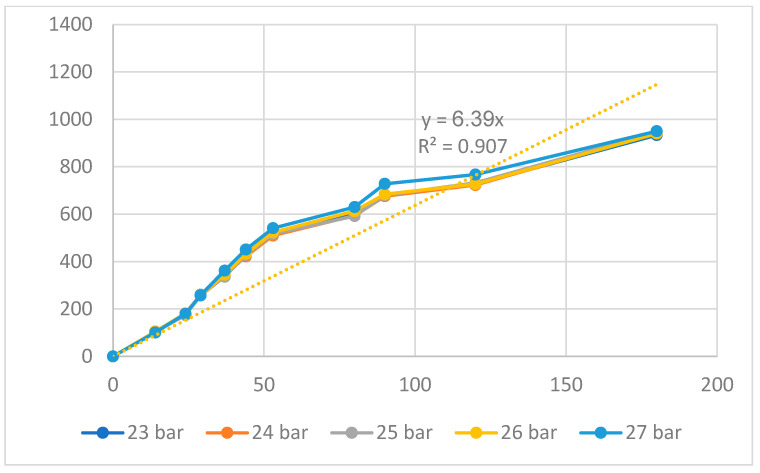
Displacement of PP flexure hinges at different packing pressures.

Figure 14 and Figure 15 show the displacement of the PP flexure hinge at different packing times and packing pressures. The regression equations between the average values of the input and the output displacements of the PP flexure hinge are

*y*_11_ = 5.08*x*_11_, (11)

*y*_12_ = 6.39*x*_12_, (12)

where *y*_11_ and *y*_12_ are the output data and *x*_11_ and *x*_12_ are the input data of the PP flexure hinge at different packing times and packing pressures.

The correlations between these factors are linear. The amplification ratio values for the packing time and packing pressure situations are 5.08 and 6.39, respectively. Because of the lower elastic modulus and tensile strength, these ratios are significantly lower than those of the ABS flexure hinge, which are 7.68 and 8.08.

**Figure 16:**
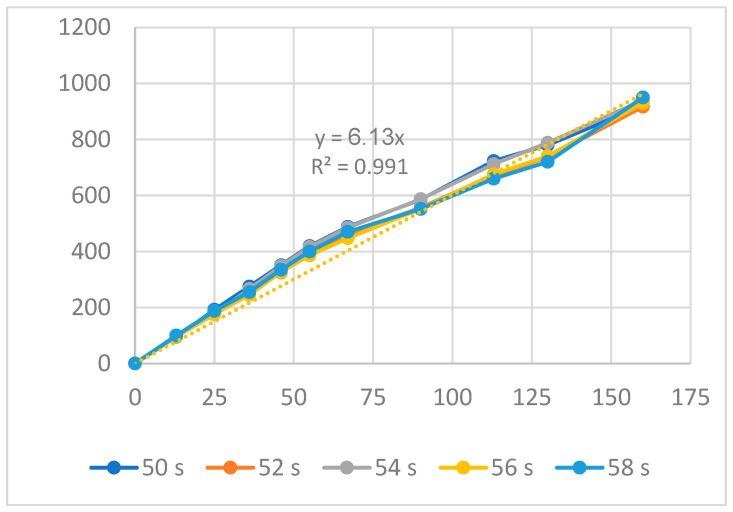
Displacement of PP flexure hinges at different cooling times.

**Figure 17:**
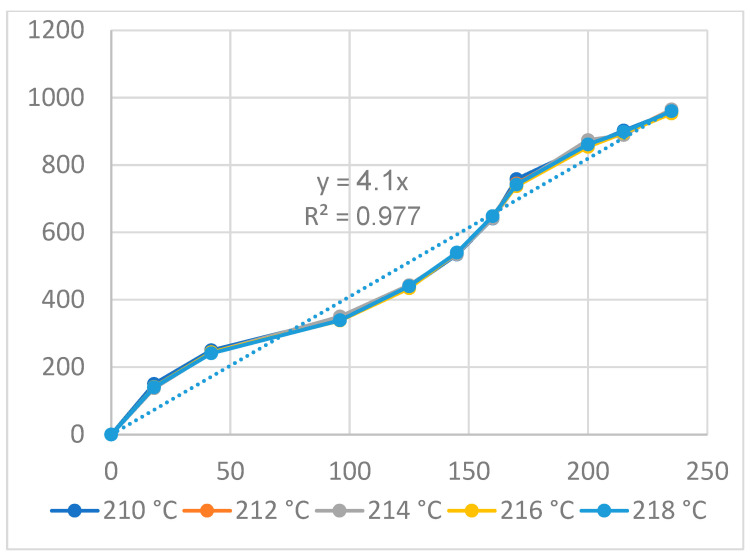
Displacement of PP flexure hinges at different melt temperatures.

Figure 16 and Figure 17 display the displacement of the PP flexure hinge at different cooling times and melt temperatures. The curve diagrams of the various parameters in each figure are most comparable, demonstrating that changing the cooling time and melt temperature has little effect on the displacement rate. Furthermore, the regression equations between the average values of the input and output displacements are as follows:

*y*_13_ = 6.13*x*_13_, (13)

*y*_14_ = 4.1*x*_14_, (14)

where *y*_13_ and *y*_14_ are the output data and *x*_13_ and *x*_14_ are the input data of the PP flexure hinge at different cooling times and melt temperatures.

The amplification ratio values for the cooling time and melt temperature cases are 6.13 and 4.1, respectively. The PP flexure hinge has lower amplification ratios than the ABS flexure hinge, which has values of 6.92 and 6.47 due to its lower elastic modulus and tensile strength.

Overall, the amplification ratio values are 6.73, 4.58, 4.89, 5.08, 6.39, 6.13, and 4.1, corresponding to the filling time, filling pressure, filling speed, packing time, packing pressure, cooling time, and melt temperature. The filling time achieves the greatest amplification ratio, while the melt temperature has the lowest one. Notably, these values are mainly lower than the ABS flexure hinge because of the weaker strength. The amplification ratio of the ABS flexure hinge could range from 4.81 to 8.81, while the amplification ratio of the PP flexure hinge could range from 4.1 to 6.73. The suitable amplification ratio could be achieved using two methods: changing the plastic-type or changing the injection parameters. In the next section, the displacement of the HDPE flexure hinge is investigated and then compared to the PP and ABS flexure hinge. Furthermore, the PP flexure hinge’s linear relationship between input and output data is similar to the ABS one.

In general, the amplification ratio of the PP flexure hinge varies in the range of 4.1–6.73. Compared to the amplification ratio of the ABS hinge, the amplification ratio of the PP flexure hinge is slightly lower. Moreover, compared to the Na et al. [38] report, the similar steel flexure hinge has an amplification of 16.2. The amplification ratio of the ABS hinge is lower due to the lower strength and stiffness of the PP plastic compared to the steel material.

### 3.3. HDPE Flexure Hinge and Comparison

**Figure 18:**
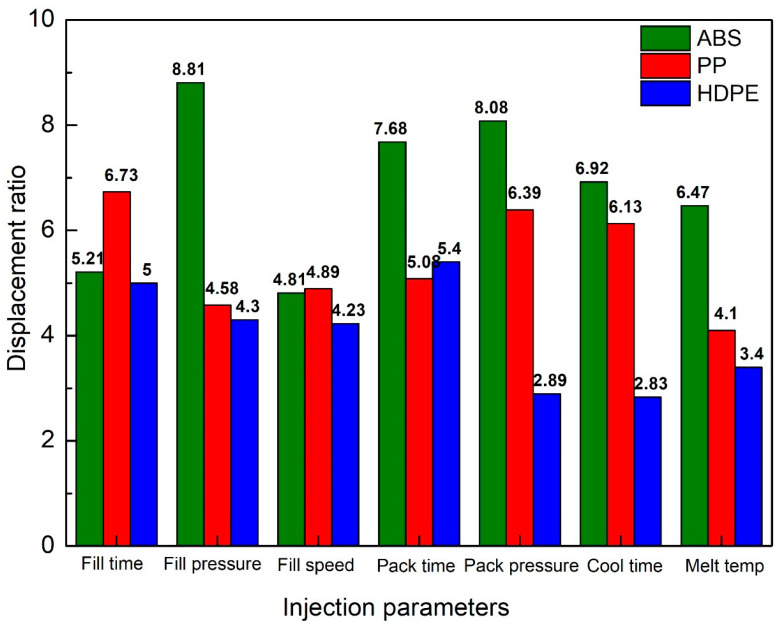
Comparison of displacement ratio among ABS, PP, and HDPE flexure hinges.

In this section, displacements of the HDPE flexure hinge are surveyed. After that, these data are compared with the previous results of the ABS and PP flexure hinges. The input and output equations at the different filling times, filling pressures, filling speeds, packing times, packing pressures, cooling times, and melt temperatures are presented in Table 3. Figure 18 shows a more visual representation of these results.

Figure 18 shows the comparison of the amplification ratio among ABS, PP, and HDPE flexure hinges. The average amplification ratio values of all injection molding parameters are 6.85, 5.41, and 4.01, corresponding to ABS, PP, and HDPE flexure hinges. Generally, the ABS flexure hinge has the highest amplification ratios, followed by the PP flexure hinge. HDPE flexure hinge has the lowest amplification ratios among these plastic types. The reason is this order’s gradual reduction in the tensile strength and elastic modulus [39]. Moreover, the highest amplification ratio is 8.81, gained by the ABS flexure hinge in the packing pressure case. In reverse, the HDPE flexure hinge in the cooling time case has the lowest amplification ratio of 2.83. The mechanical properties strongly affect the performance of the plastic flexure hinge. The flexure hinge shape, in this case, is not suitable for finding Young’s modulus, as it is not a tensile test sample. The shape of the flexure hinge and the process parameters could impact Young’s modulus value via the degree of crystallinity. The Young’s modulus of ABS, PP, and HDPE are 3110 MPa, 1550 Mpa, and 1300 Mpa, respectively. These values are very useful to explain the average amplification ratio values of these plastic hinges. For ABS, PP, and HDPE flexure hinges, the average amplification ratio values of all injection molding parameters are 6.85, 5.41, and 4.01. Therefore, the higher the Young’s modulus value, the higher the amplification ratio value of the plastic hinges.

The optimal function here is the maximum amplification ratio, which is the “a” factor” in the equation y = a.*x*. This study tries to find the injection parameters that generate the maximum amplification ratio. In addition, the optimal injection parameter for the ABS flexure hinge with the highest amplification ratio is a filling time of 2.4 s, a filling pressure of 39–43 bar, a filling speed of 79%, a packing time of 0.6 s, a packing pressure of 40 bar, a cooling time of 24 s, and a melt temperature of 210 °C. With these optimal injection parameters and the input value of 105 µm, the ABS flexure hinge could create a maximum average output value of 736.6 µm. The optimal injection parameter for the PP flexure hinge is a filling time of 1.5–2.3 s, a filling pressure of 27 bar, a filling speed of 54%, a packing time of 0.6 s, a packing pressure of 25 bar, a cooling time of 24 s, and a melt temperature of 214 °C. With the optimal injection parameter, and the input value of 128 µm, the PP flexure hinge could create a maximum average output value of 964.8 µm. The optimal injection parameter for the HDPE flexure hinge is like the PP hinge, which is a filling time of 1.5–2.3 s, a filling pressure of 27 bar, a filling speed of 54%, a packing time of 0.6 s, a packing pressure of 25 bar, a cooling time of 24 s, and a melt temperature of 214 °C. With the optimal injection parameter, and the input value of 218 µm, the HDPE flexure hinge could create a maximum average output value of 699.8 µm.

Table 4 presents the amplification ratios of different flexure hinge materials. The aluminum alloys and steel alloys could produce a flexure hinge with a greater amplification ratio compared to the ABS, PP, and HDPE flexure hinges [37,38]. However, the amplification ratios of the ABS, PP, and HDPE flexure hinges are compatible with smart memory alloys and titanium alloys [40,41]. Most importantly, the injection plastic hinges may be mass-produced at a low cost and high productivity level. On the other hand, the cost and difficulty of producing the metallic flexure hinges are significantly higher than those of injection plastic ones. Therefore, the injection plastic hinges could facilitate the application of flexural hinges. In the future, we could examine how different hinge forms and corner shapes affect the functionalities of injection flexure plastic hinges. The fatigue strength of the injection plastic hinge could also be investigated.

## 4. Conclusions

This study investigates the amplification of the injected plastic flexure hinge. The effects of injection plastics and injection parameters on the amplification ratio of the compliant mechanism flexural hinges are surveyed. The flexural hinge is injected with different plastic types: filling time, filling pressure, filling speed, packing time, packing pressure, cooling time, and melt temperature. Some noteworthy remarks that may be noted include the following:

The injection plastic hinges could be produced at the mass production level with high productivity and low cost. The relationship between the input and output data of the ABS, PP, and HDPE flexure hinges at different injection molding parameters is a linear relation. Changing the hinge’s material or numerous injection molding settings could have a significant impact on its performance.

With the ABS flexure hinge, the packing pressure case has the greatest amplification ratio of 8.81, while the filling speed case has the lowest value of 4.81. The optimal injection parameter for an ABS flexure hinge is a filling time of 2.4 s, a filling pressure of 41 bar, a filling speed of 79%, a packing time of 0.6 s, a packing pressure of 38–42 bar, a cooling time of 24 s, and a melt temperature of 210 °C. The ABS flexure hinge could produce a maximum average output value of 736.6 µm with this parameter and an input value of 105 µm.

The filling time case has the highest amplification ratio of 6.73 with the PP flexure hinge, while the melt temperature case has the lowest value of 4.1. The optimal injection parameter for a PP flexure hinge is a filling time of 1.5–2.3 s, a filling pressure of 27 bar, a filling speed of 54%, a packing time of 0.6 s, a packing pressure of 25 bar, a cooling time of 24 s, and a melt temperature of 214 °C. With this parameter, and the input value of 128 µm, the PP flexure hinge could create a maximum average output value of 964.8 µm.

With the HDPE flexure hinge, the packing time case achieves the greatest amplification ratio of 5.4, while the cooling case has the lowest value of 2.83. The optimal injection parameter for the HDPE flexure hinge is like the PP hinge, which is a filling time of 1.5–2.3 s, a filling pressure of 27 bar, a filling speed of 54%, a packing time of 0.6 s, a packing pressure of 25 bar, a cooling time of 24 s, and a melt temperature of 214 °C. The largest average output value that the HDPE flexure hinge could produce with the ideal injection parameter and an input value of 218 µm is 699.8 µm.

For the ABS, PP, and HDPE flexure hinges, the amplification ratio values of all injection molding parameters are 4.81–8.81, 4.1–6.73, and 2.83–5.4. In general, the ABS flexure hinge could be superior to the PP flexure hinge in terms of amplification ratios. The lowest amplification ratio among these plastic kinds is found in HDPE flexure hinges. The results provide more insight into plastic flexure hinges and broaden their applications by finding the optimal injection parameters and plastic types. In the future, we could analyze the effects of corner shapes and try other hinge shapes on the performance of the flexure plastic hinges.

## Figures and Tables

**Table 1:** Injection molding parameters of ABS hinges.

Group	Filling Time (s)	Filling Pressure (bar)	Packing Time (s)	Packing Pressure (bar)	Cooling Time (s)	Melt Temperature (°C)	Filling Speed (%)
	2						
	2.2						
1	2.4						
	2.6	41	0.6	40	24	210	79
	2.8						
		39					
		40					
		41					
2	2.4	42	0.6	40	24	210	79
		43					
			0				
			0.3				
			0.6				
3	2.4	41	0.9	40	24	210	79
			1.2				
				38			
				39			
				40			
4	2.4	41	0.6	41	24	210	79
				42			
					20		
					22		
					24		
5	2.4	41	0.6	40	26	210	79
					28		
						206	
						208	
						210	
6	2.4	41	0.6	40	24	212	79
						214	
							75
							77
							79
7	2.4	41	0.6	40	24	210	81
							83

**Table 2:** Injection molding parameters of HDPE and PP hinges.

Group	Filling Time (s)	Filling Pressure (bar)	Packing Time (s)	Packing Pressure (bar)	Cooling Time (s)	Melt Temperature (°C)	Filling Speed (%)
	1.5						
	1.7						
	1.9						
1	2.1	27	0.6	25	54	214	54
	2.3						
		25					
		26					
		27					
2	1.9	28	0.6	25	54	214	54
		29					
			0				
			0.3				
			0.6				
3	1.9	41	0.9	25	54	214	54
			1.2				
				23			
				24			
				25			
4	1.9	41	0.6	26	54	214	54
				27			
					50		
					52		
					54		
5	1.9	41	0.6	25	56	214	54
					58		
						210	
						212	
						214	
6	1.9	41	0.6	25	54	216	54
						218	
							50
							52
							54
7	1.9	41	0.6	25	54	214	56
							58

**Table 3:** Displacement equations of the HDPE flexure hinge.

Injection Parameters	Equations
Filling time	*y*_15_ = 5.0*x*_15_; R^2^ = 0.938
Filling pressure	*y*_16_ = 4.33*x*_16_; R^2^ = 0.967
Filling speed	*y*_17_ = 4.23*x*_17_; R^2^ = 0.988
Packing time	*y*_18_ = 5.4*x*_18_; R^2^ = 0.965
Packing pressure	*y*_19_ = 2.89*x*_19_; R^2^ = 0.952
Cooling time	*y*_20_ = 2.83*x*_20_; R^2^ = 0.942
Melt temperature	*y*_21_ = 3.4*x*_21_; R^2^ = 0.993

**Table 4:** Amplification ratios of different flexure hinge materials.

Materials	Amplification Ratio	References
ABS	5.01–8.15	This study
PP	3.99–7.9	This study
HDPE	2.17–6.24	This study
Aluminum alloys	5–25	Kim et al. [37]
Steel alloys	16.2	Na et al. [38]
Smart memory alloys	2.2	Maffiodo et al. [40]
Titanium alloys	6.0	Fiaz et al. [41]

## Data Availability

The data used to support the findings of this study are available from the corresponding author upon request.
